# Study of copper-cysteamine based X-ray induced photodynamic therapy and its effects on cancer cell proliferation and migration in a clinical mimic setting

**DOI:** 10.1016/j.bioactmat.2021.05.016

**Published:** 2021-05-30

**Authors:** Xiangyu Chen, Jiayi Liu, Ya Li, Nil Kanatha Pandey, Taili Chen, Lingyun Wang, Eric Horacio Amador, Weijun Chen, Feiyue Liu, Enhua Xiao, Wei Chen

**Affiliations:** a Department of Radiology, The Second Xiangya Hospital of Central South University, Changsha, Hunan, China; b Department of Physics, The University of Texas at Arlington, Arlington, TX, 76019, USA; c Department of Oncology, Xiangya Hospital, Central South University, Changsha, Hunan Province, 410011, China; d Department of Oncology, The Second Xiangya Hospital of Central South University, Changsha, Hunan, China

**Keywords:** Copper-cysteamine, Nanoparticle, Photodynamic therapy, X-ray, Cell migration, Proliferation

## Abstract

Copper-cysteamine as a new generation of sensitizers can be activated by light, X-rays, microwaves, or ultrasound to produce reactive oxygen species. X-ray induced photodynamic therapy (X-PDT) has been studied extensively; however, most of the studies reported so far were conducted in the laboratory, which is not conducive to the clinical translation conditions. In this contribution, for the first time, we investigated the treatment efficiency of copper-cysteamine (Cu-Cy) based X-PDT by mimicking the clinical conditions with a clinical linear accelerator and building deep-seated tumor models to study not only the effectiveness but also its effects on the cell migration and proliferation in the level of the cell, tissue, and animal. The results showed that, without X-ray irradiation, Cu-Cy nanoparticles (NPs) had a low toxicity in HepG2, SK-HEP-1, Li-7, and 4T1 cells at a concentration below 100 mg/L. Interestingly, for the first time, it was observed that Cu-Cy mediated X-PDT can inhibit the proliferation and migration of these cell lines in a dose-dependent manner. Antigen markers of migration and cell proliferation, proliferating cell nuclear antigen (PCNA) and E-cadherin, from tumor tissue in the X-PDT group were remarkably different from that of the control group. Furthermore, the MRI assessment showed that the Cu-Cy based X-PDT inhibited the growth of deeply located tumors in mice and rabbits (*p* < 0.05) without any obvious toxicities *in vivo*. Overall, these new findings demonstrate that Cu-Cy NPs have a safe and promising clinical application prospect in X-PDT to improve the efficiency of radiotherapy (RT) for deep-seated tumors and effectively inhibit tumor cell proliferation and migration.

## Highlights

• X-PDT was studied by mimicking the clinical conditions with a clinical linear accelerator and building deep-seated tumor.
• Cu-Cy mediated X-PDT can inhibit the proliferation and migration of these cell lines in a dose-dependent manner.
• Cu-Cy based X-PDT inhibited the growth of deeply located tumors in mice and rabbits without any obvious toxicities *in vivo*.
• Cu-Cy NPs have a safe and promising clinical application prospect in X-PDT to improve the efficiency of radiotherapy.

## Graphical abstract

**Figure:**
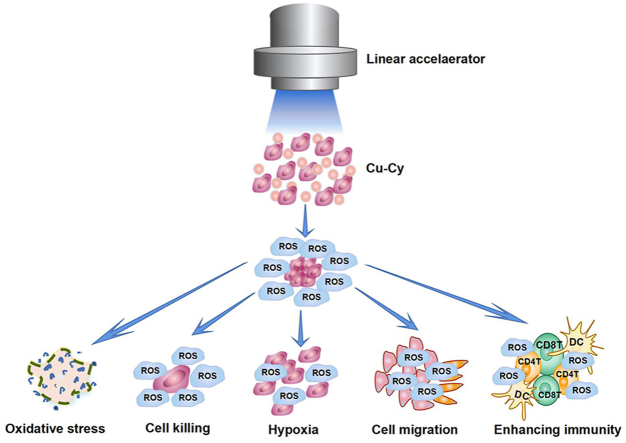

## 1 Introduction

Cancer is one of the leading causes of death around the world [1]. In the clinical field, doctors use different methods to fight against cancers, such as surgery, chemotherapy, and radiotherapy (RT). As we all know, surgery and chemotherapy are painful for patients. Although RT effectively inhibits various tumor cell phenotypes to damage tumor cells fatally, it causes non-specific damage to normal tissues [2]. For example, to destroy deep-seated tumors, a high dose of X-rays (over 5 Gy at a time) is needed, which increase the DNA damage in healthy tissue close to the tumor. Hence, it is essential to control the dose of radiation to improve the efficacy of RT.

With the development of nanotechnology, photodynamic therapy (PDT) has emerged as an efficient treatment method for cancer patients with many advantages, including fewer side effects to normal tissues and high selectivity [[3], [4], [5]]. Nevertheless, ultraviolet (UV), visible light or near-infrared (NIR) light-induced PDT has the characteristics of low tissue penetration and rapid energy decay. Consequently, the efficacy of tumor eradication reduces if the tumors are located in deep tissue [6,7]. In order to solve these problems, researchers have proposed the concept of X-ray induced photodynamic therapy (X-PDT) [8,9], which uses an energy transducer in order to transfer X-rays to initiate the RT and PDT processes [3]. Until recently, X-PDT has undergone a long period of development and shown less irradiation and good efficacy *in vitro* and in *vivo*. Due to the synergistic effect of PDT and RT, X-PDT has overcome the limitations of traditional therapies and owned a good perspective of biomedical applications [3]. However, most of the experimental studies were conducted in laboratories, which is not conducive to the clinical translation of X-PDT. Consequently, it is highly desirable to investigate the benefits and drawbacks of X-PDT in a clinical environment.

Copper-cysteamine (Cu-Cy) NPs are promising X-ray induced photosensitizing agents, which produce singlet oxygen for efficient cancer treatment [10,11]. Notably, Cu-Cy NPs have a striking characteristic compared with other nanoparticle radiosensitizers, because it can act as a photosensitizer to produce ROS directly induced by X-ray [[11], [12], [13], [14], [15]] instead of transferring the absorbed energy from X-ray to other NPs [8,9,16]. Previous studies also demonstrate that Cu-Cy NPs have several important applications and tremendous potential for cancer therapy *in vitro* or with subcutaneous tumor models *in vivo* under laboratory environment when activated by UV [10,17], X-ray [[11], [12], [13], [14], [15]], microwaves [6,18], or ultrasound [19]. Here, for the first time, we performed the study of Cu-Cy nanoparticles using deeply-located models to mimic the clinical conditions by a clinical linear accelerator with a lower RT dose in order to comprehensively understand the real-world biomedical applications of Cu-Cy NPs in different biological levels. Moreover, we attempted to elucidate the mechanism of Cu-Cy NPs mediated X-PDT not only observing their efficacies on cancer cell killing and tumor-shrinking but also their effects on cancer cell migration and proliferation, and for the first time, we discovered that Cu-Cy mediated X-PDT can inhibit the proliferation and migration of cancer cell lines in a dose-dependent manner and that the Cu-Cy based X-PDT inhibited the growth of deeply located tumors in mice and rabbits without any obvious toxicities *in vivo*.

## 2 Materials and methods

### 2.1 Synthesis and characterizations of Cu-Cy NPs

Cu-Cy NPs were fabricated using the previously reported protocol [10,18]. The UV–vis absorption spectra were obtained using a UV–Vis spectrophotometer (Shimadzu UV-2450, Japan) and the photoluminescence emission and excitation spectra were collected using a spectrofluorophotometer (Shimadzu RF-5301PC, Japan). TEM images of the Cu-Cy NPs were obtained using a transmission electron microscope (JEM-2100 HR, JEOL Ltd, Japan).

### 2.2 Cell culture

The cell lines involved in this work (HepG2, SK-HEP-1, Li-7, and 4T1) were received from the Cell Bank of the Chinese Academy of Sciences (Shanghai, China). These cell lines were immediately proliferated and frozen. When we started different parts of the experiments, they were used from a frozen tubule of the same batch of the cell line. These cells were cultured in Roswell Park Memorial Institute (RPMI) medium or Dulbecco's Modified Eagle Medium (DMEM) medium (Hyclone, GE Healthcare Life Sciences, Utah, USA), 10% FBS (Biological Industries, Beit haemek, Israel), and 5% penicillin-streptomycin (Hyclone, GE Healthcare Life Sciences, Utah, USA) with culture conditions of 37 °C with 5% CO_2_.

### 2.3 CCK8 assay

The CCK8 assay was studied using HepG2, SK-HEP-1, Li-7, and 4T1 cells. On the one hand, these cell lines were seeded in a 96-well plate at a density of 1 × 10^4^ cells/mL and cultured by 0, 10, 25, 50, 100, or 150 mg/L of Cu-Cy NPs for 24 h in the dark. On the other hand, the cells were also seeded and cultured by the same steps as mentioned above for 6 h in the dark, separately. Afterward, the cells were covered by a layer of pork (around 2 cm thickness) between a culture dish and an X-ray generator and irradiated by a clinical linear accelerator (Varian Trilogy, CA, USA) at 6 MV, 100 MU/min for 2 Gy. After irradiating, the cell lines were incubated for another 24 h at 37 °C with 5% CO_2_. The cell viability was measured by a Cell Counting Kit-8 assay (CCK-8, Beyotime, Shanghai, China). 10 μL of the CCK-8 solution and 90 μL of media were added to each well. The absorbance (450 nm) for each well was then monitored by a microplate reader (Infinite 200 pro, TECAN, Shanghai, China) after incubating for the same time interval at every plate.

The Initiatory time of cell culture is the same in different groups. Although 2Gy group was received by radiation therapy, the OD values of 2Gy group and Cu-Cy group were also tested at the same time.

To mimic the antitumor efficacy in a deep location, we used a layer of pork (around 2 cm thickness), the fresh or salted flesh of swine, which covered the surface of the 96-well plate in CCK8 assay. We put a pork layer on because the tissue density of pork is equivalent to that of human tissue. Pork of appropriate thickness can simulate the attenuation of rays passing through the human body, thereby simulating the killing effect on cells in deep tissues as described in literature [20].

### 2.4 Transwell assay

The cell migration assessment of Cu-Cy NPs was investigated using HepG2 and SK-HEP-1 cells. When the cells covered approximately 60% of the bottom of the T25 culture bottle, 5 mL of Cu-Cy NPs with different concentrations (0, 50, and 100 mg/L) with, which was diluted with complete media, were added to the culture bottle. The cells were covered by a layer of pork and were irradiated by a clinical linear accelerator in the manner mentioned above and incubated for another 24 h at 37 °C with 5% CO_2_. Next, the upper Transwell chamber was pre-coated with Matrigel (BD Bioscience, Shanghai, China). The cells after different treatments with the same number around 2 × 10^4^ were seeded onto the upper Transwell chamber with a serum-free media. The complete media with 10% serum was added to the lower chamber. After the migration of cells for 24 h, the cells were fixed with 4% paraformaldehyde, then stained with Wright-Giemsa solution, and finally counted under a light microscope.

### 2.5 Animal models

All animal works were performed following the protocols of the Central South University of Second Xiangya Hospital Animal Care Committee (SYXK-2017-0002). 6–8 weeks female BALB/c nude mice and New Zealand white rabbits of both sexes weighing between 2.5 kg and 3.0 kg were ordered from the Experimental Animal Center of the Second Xiangya Hospital of Central South University. The breast cancer mouse model was established using nude mice by injecting subcutaneously into the back of the mouse with 100 μL of 4T1 cells (around 2 × 10^6^ cells). The New Zealand white rabbits were inoculated with 0.5 mL of VX2 tumor fragments in the liver through the methods of inhalation anesthesia and ultrasound-guided implantation to form an in situ tumor model of hepatocarcinoma [21].

The VX2 orthotopic hepatocarcinoma grows very fast, so we chose the 7 days as an observational cycle. The collected tumors were cut into small pieces (0.5–1 mm diameter) and stored in phosphate-buffered solution. Subsequently, the tumor tissue fragments were drawn up with a 1-m syringe and injected into the recipient rabbit's liver. Then, the entire liver was observed using an abdominal ultrasound to evade important blood vessels and to confirm the thickest area or edge between the two left lobes of the liver as the target implantation site. Next, a 16 G hollow needle connected to a syringe filled with approximately 0.5 ml of tumor fragments was inserted into the predetermined location under ultrasound guidance and the tumor fragments in the syringe were injected.

### 2.6 *In vivo* treatment and safety assessment

When the size of subcutaneous 4T1 tumors reached about 5 mm in diameter, mice were randomly separated into four groups (n = 5 for each group): Saline, X-ray, Cu-Cy, and X-PDT groups and were performed via the different interventional methods once in two days before the end of the treatment. In the control group, 50 μL of saline was injected intratumorally into the tumor. In the Cu-Cy and X-PDT groups, 50 μL of Cu-Cy NPs (1 μg/μL) were injected intratumorally into the tumor. After 30 min post-injection, the mice in the X-ray and X-PDT groups were covered by a layer of pork as mentioned above and irradiated by a clinical linear accelerator (Varian Trilogy, CA, USA) at 6 MV, 100 MU/min for 2 Gy.

On the third day after VX2 tumor implantation, rabbits were randomly divided into four groups (n = 3, for each group) as mentioned above, and different interventional treatments were applied via US-guided intra-tumor injection once a day until the seventh day of implantation. In the control group, 3 mL of saline was injected. In the Cu-Cy and X-PDT groups, 3 mL of Cu-Cy NPs (1 mg/mL) were injected. After 30 min post-injection, the rabbits in the X-ray and X-PDT groups were irradiated by a clinical linear accelerator (Varian Trilogy, CA, USA) at 6 MV, 100 MU/min for 2 Gy. All animals in the process of the experiment were observed for death or abnormal behavior. Tumor volume was assessed by an MRI system, as shown below. Animal body weight was measured by an electronic scale under a regular time interval.

After 3 days of implantation, we could see the shape of the tumor by ultrasonic machine and perform intra-tumor injection once a day from 3rd until 7th day by percutaneous puncture under ultrasound guidance so that the liquid accumulated in the tumor as possible. Due to the short cycle of observation, repeated MRI examination or anesthesia could influence animal welfare and maybe even study results, leading to an interpretation bias.

### 2.7 MR assessment and verification

The images of the mice's subcutaneous transplantation were recorded before the treatment and every 5 days after the treatment. The images of rabbit liver VX2 hepatocarcinoma were recorded on the seventh day after implantation. All imaging was performed using a 3.0 T MRI system (Siemens Skyra, München, Germany) with a 15 Ch phased-array torso coil (MDSS GmbH, Hannover, Germany) and a 4 Ch mouse coil (Medcoil Healthcare, Suzhou, China). The MRI sequences included a T1-weighted fast gradient-echo sequence and a T2-weighted fast spin-echo sequence. Tumor volume was measured by semi-automatic 3D volume segmentation software (Siemens Syngo, München, Germany). An experienced radiologist (10 years of experience), who did not perform the implantation procedures, delineated the regions of interest (ROI) of the tumor at every slice in the axial view, built a 3D tumor segmentation model, and finally calculated the final volume.

### 2.8 Histopathology and immunohistochemistry examination

The mice were euthanized on the tenth day after different interventions. Afterward, vital organs (heart, liver, spleen, lung, and kidneys) and tumors from different groups were fixed in a 10% formalin solution within 24 h, paraffin sectioned, and followed by staining with hematoxylin-eosin (H&E). Also, immunohistochemistry was performed to evaluate the invasion/metastatic ability of tumor after treatment. Brieﬂy, paraffin-embedded tumor sections were dewaxed and hydrated using ethanol with various concentrations and blocked with 5% FBS. Then, sections were incubated with anti-E-cadherin and anti-PCNA antibodies (Beyotime Institute of Biotechnology, Shanghai, China), and followed by incubation with the secondary antibody (Zhongshan Golden Bridge, Beijing, China). Next, the sections were developed in diaminobenzidine (DAP) solution, counterstained with hematoxylin, dehydrated in gradient alcohol, and sealed in resins. The results of HE, E-cadherin, and PCNA were observed and recorded under a microscope with the similar light condition. The morphological changes were observed by an experienced pathologist (5 years of experience), and the percentages of the expression of E-cadherin and PCNA were evaluated in these three different hotspot fields of view using ImageJ software (version 1.52, MD, USA).

The tumor and organs were large, and the slices were small. We randomly chose the fragments of vital organs and tumors to fix and section. Then, we repeated experiments at least 3 times to validate the repeatability of experimental results.

### 2.9 Statistical analyses

All the experiments were performed at least three times independently. Comparisons among different interventional groups were performed using the Student t-test for parametric data or the Mann-Whitney *U* test for non-parametric data. Statistical analyses were carried out using GraphPad Prism 7.0. *p*-values less than 0.05 were taken as statistically significant.

## 3 Results

### 3.1 Characterization of Cu-Cy NPs

**Fig. 1:**
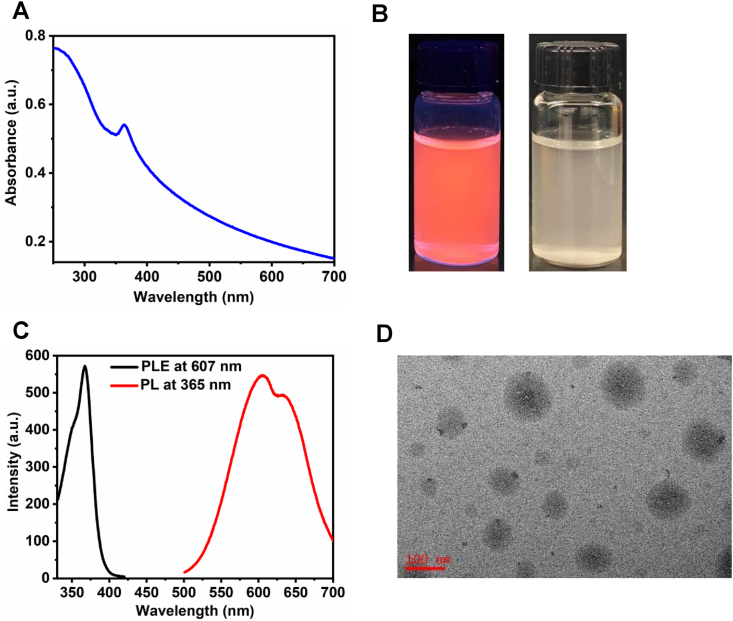
(**A)** UV–vis absorption spectrum of the Cu-Cy NPs suspended in DI water. (**B)** Pictures of the Cu-Cy NPs dispersed in DI water upon 365 nm UV light (left) and room light (right). (**C)** The spectra of photoluminescence excitation (PLE, left) at 607 nm and emission (PL, right) at 365 nm of the Cu-Cy NPs dispersed in DI water. (**D)** A representative TEM image of the Cu-Cy NPs.

**Fig. 2:**
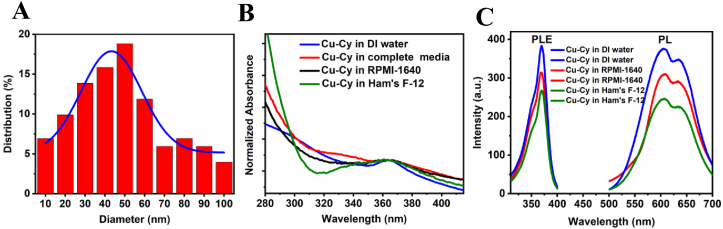
(A) Histogram of the size distribution of Cu-Cy nanoparticles used in this study. Average diameter = 43 ± 10 nm. (B) Comparison of UV–visible absorption spectra of Cu-Cy in DI water, complete cell culture media of KYSE-30 cancer cells, RPMI-1640 medium, and Ham's F-12 medium. (C) Comparison of photoluminescence excitation (PLE) and emission (PL) spectra of Cu-Cy in DI water, RPMI-1640 medium, and Ham's F-12 medium.

The detailed synthesis procedure, characterizations, and crystal-structure of Cu-Cy NPs were discussed in our previous publications [10,18]. Fig. 1A depicts the UV–vis absorption curve of the Cu-Cy NPs suspended in deionized (DI) water, with the absorption peak at about 365 nm. Cu-Cy NPs display intense photoluminescence under UV light irradiation. Fig. 1B shows the images of the Cu-Cy NPs in DI water under UV light (left) and room light (right). The photoluminescence (PL) emission spectrum (red) upon 365 nm excitation and photoluminescence excitation (PLE) spectrum (black) under 607 nm emission of the Cu-Cy NPs are displayed in Fig. 1C. These NPs show orange-red emission peaking at about 607 nm with a shoulder at about 633 nm, which are from the transitions (d^9^4s^1^ - d^10^) of the two kinds of copper ions- Cu(1) and Cu(2) in the Cu-Cy crystal that are different from each other by different coordination environment [10]. Fig. 1D shows the representative TEM image of the Cu-Cy NPs used in this contribution. Their sizes are ranging from about 10 to 100 nm, with an average diameter of approximately 40 nm, as observed from the TEM image. The particle size distribution is shown in Fig. 2A. The particle size and size-distribution are key parameters to determine the particle optical absorption peak and luminescence peak. On the contrary, the optical absorption and luminescence properties can reflect the size and distribution [22,23]. In order to study the particles size and distribution under physiological conditions, the Cu-Cy nanoparticles were dispersed in various cell-culture media and their UV–visible, PLE and PL spectra were monitored. In our observations, we found that the absorption peak as well as the emission and excitation peaks are almost the same (Fig. 2B–C), indicating that the particle size and size-distribution are the almost same or similar in physiological conditions.

### 3.2 X-PDT inhibited the cell proliferation and migration *in vitro*

**Fig. 3:**
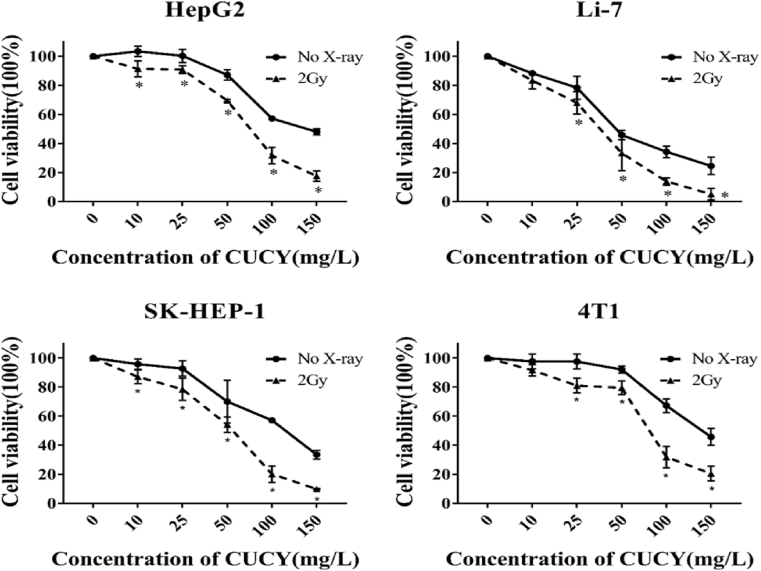
X-PDT inhibited the proliferation of tumor cells *in vitro*. The cell viability of HepG2, SK-HEP-1, Li-7, or 4T1 cells were calculated by CCK8 assay after treating 0, 10, 25, 50, 100, or 150 mg/L of Cu-Cy NPs with or without X-rays irradiation with a dose of 2 Gy *in vitro* (*p < 0.05 vs no X-ray group).

To evaluate the effectiveness of the Cu-Cy NPs mediated X-PDT for inhibiting the cell proliferation and migration, CCK8 and Transwell assays were performed using SK-HEP-1, HepG2, Li-7, or 4T1 cells. The results of the CCK8 assay (Fig. 3) showed that HepG2 and SK-HEP-1 cell lines were more sensitive to X-PDT than Li-7 cell line at 10 mg/mL of Cu-Cy NPs. However, with the rise of the concentration, SK-HEP-1, and Li-7 cells’ viability decreased to 9.8% and 5.1%, which were less than the viability for HepG2 cells with 17.5% and 4T1 cells with 20% at a Cu-Cy NPs concentration of 150 mg/L after X-PDT. The cell viability of 4T1 also rapidly decreased after X-PDT as the Cu-Cy concentration increased from 25 mg/L to 150 mg/L (*p* < 0.05). The results suggested that Cu-Cy NPs had low dark cytotoxicity in most cells at a concentration below 100 mg/L. Accordingly, 100 mg/L as an upper-limit concentration was used for further experiments *in vitro* to avoid the intrinsic toxicity from the NPs themselves.

**Fig. 4:**
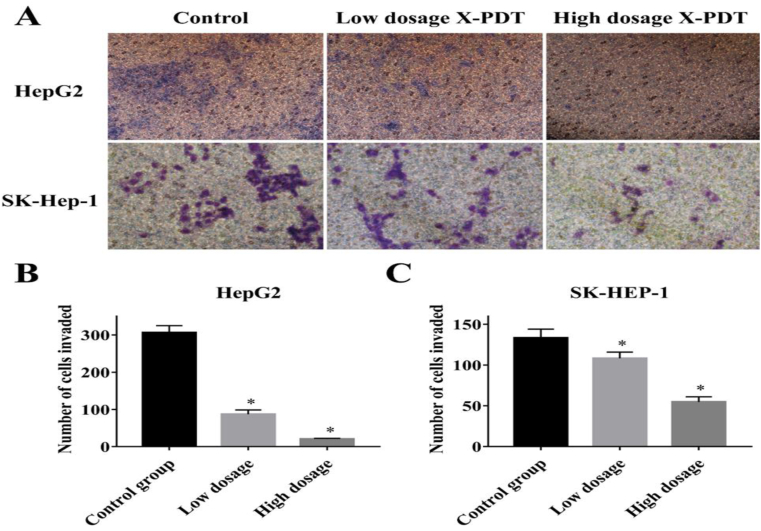
X-PDT inhibited the migration of tumor cells *in vitro*. (**A)** Transwell assays detected the migration of HepG2 and SK-Hep-1 after the X-PDT with low dosage (50 mg/L) and high dosage (100 mg/L) of Cu–Cy NPs, separately. (**B–C)** The number of migratory cells was counted and plotted on two graphs (*p < 0.05 vs control group).

Then, changes in the cell migration capacity in response to X-PDT were assessed by the Transwell assay. The results (Fig. 4A) showed that the number of invaded cells at low and high concentrations of the X-PDT group decreased significantly when compared to the control. As displayed in Fig. 4B and C, the number of HepG2 cells in the control group was 3.5-fold and 15.3-fold higher than that in the low concentration X-PDT group (*p* < 0.05) and in high concentration X-PDT group (*p* < 0.05), respectively. The number of SK-HEP-1 cells in the control group was 1.23-fold and 2.4-fold higher than that in the low concentration X-PDT group (*p* < 0.05) and in high concentration X-PDT group (*p* < 0.05), respectively. In summary, these findings suggested that X-PDT inhibited cell proliferation and migration *in vitro*.

**Fig. 5:**
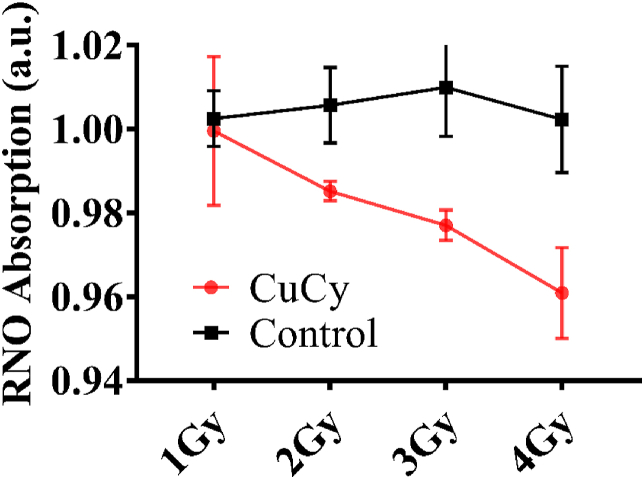
The ROS production in Cu-Cy NPs under X-ray irradiation.

Fig. 5 shows the ROS production under the X-ray irradiation used for this study. Obviously, ROS is produced under the clinic used X-rays used for this research, which provides strong support and evidence for the observations described here. It is true that X-ray alone can kill cells. However, at a very low dose of 2 Gy, we found that the cell damage by X-ray is very less. We have conducted many studies for the comparison and published in our previous papers [11,13,24,25].

The same conclusion is shown in this study as see in Fig. 3. The x-axis represents the concentrations of Cu-Cy particles. At the zero concentration of Cu-Cy as indicated by the red spots, it is the X-ray alone on the cells, and it can see clearly that the cell viability is almost 100%. This indicates further that X-ray alone at a low dose of 2 Gy cannot damage the cells in a noticeable way. However, the combination of X-ray and Cu-Cy nanoparticles are lethal to the tumor cells.

### 3.3 X-PDT inhibited the growth of tumors *in vivo*

**Fig. 6:**
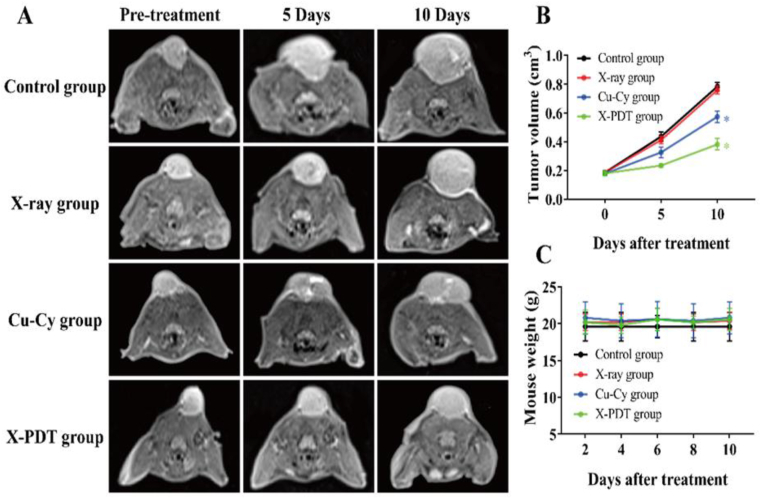
X-PDT enhanced the tumoricidal effect in mouse subcutaneous tumor models. (**A)** MR assessment of tumoricidal effect after different interventions. (**B)** Tumor volume changes (*p < 0.05 vs control group). (**C)** Body weight changes.

To investigate the effectiveness of the Cu-Cy NPs for liver and breast tumors *in vivo*, two types of animal models were used. As for the subcutaneous breast tumor model, the results of the MR assessment are shown in Fig. 6A. The quantitative changes in tumor volume are presented in Fig. 6B. Obviously, the volume of subcutaneous tumors on the mice grew in varying degrees, but the growth of tumors in the X-PDT group was the slowest with 0.27 cm^3^, which were remarkably smaller than that of the control group with 0.47 cm^3^ (*p* < 0.05). The tumor volume in the Cu-Cy group with 0.56 cm^3^ was also smaller than that of the control as mentioned above (*p* < 0.05), whereas no statistically significant difference between control and X-ray groups (*p* > 0.05) was observed.

**Fig. 7:**
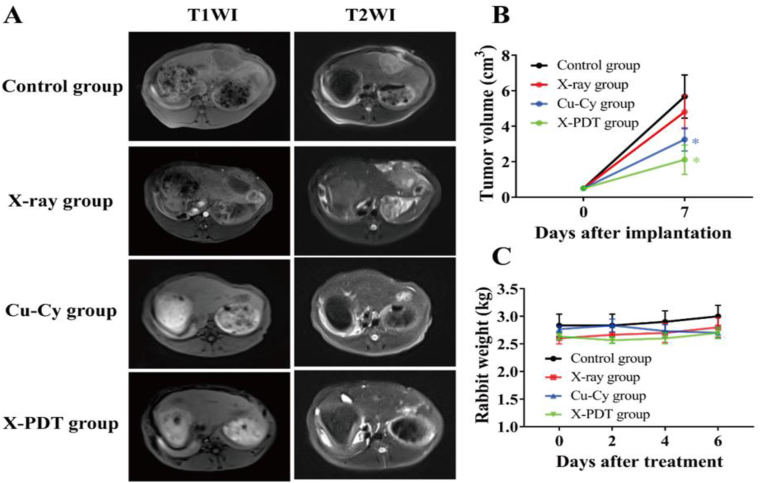
X-PDT enhanced the tumoricidal effect in rabbit VX2 tumor models. (**A**) MR assessment of tumoricidal effect after different interventions. (**B)** Tumor volume changes (*p < 0.05 vs control group). (**C)** Body weight changes.

As shown in Fig. 7A and B, the volumes of XV2 liver tumor in the X-PDT group with 1.31 cm^3^ (*p* < 0.05) and Cu-Cy group with 1.88 cm^3^ (*p* < 0.05) were smaller than that of the control group with 3.09 cm^3^, while no statistically significant difference between control and X-ray groups (*p* > 0.05) was found. The above results implied that X-PDT inhibited the growth of breast tumor and in situ liver tumor.

### 3.4 The safety and mechanism of X-PDT *in vivo*

**Fig. 8:**
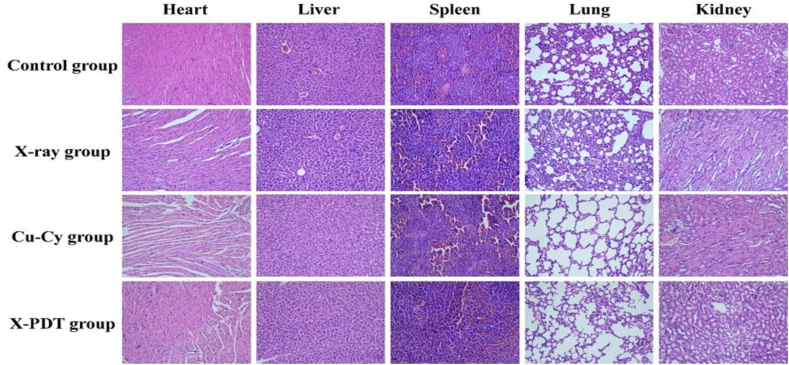
H & E staining of heart, liver, spleen, lung, and kidney at the end of treatment. H & E staining with magnification of 200×.

To indicate the safety and biocompatibility, no acute toxicity reaction occurred after the X-PDT in mice and rabbits, such as death and abnormal behavior. In addition, the body weight of the mice and rabbits were monitored on both the breast model and liver model during treatment. The X-PDT group and Cu-Cy group showed no significant weight loss (Fig. 6, Fig. 7C). Histological evaluation for major organs did not show inflammation or any metastatic tumor cell (Fig. 8). These results indicate that Cu-Cy and Cu-Cy based X-PDT treatment have a good biocompatibility and low toxicity.

**Fig. 9:**
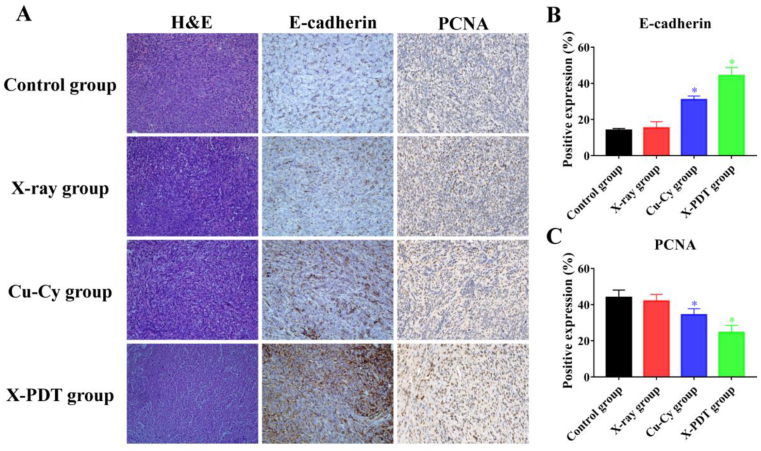
Morphological examination and immunohistochemistry of tumors. (**A)** H&E staining, E-cadherin, and PCNA immunohistochemistry staining of tumor tissues. (**B)** The percentages of the positive expression of E-cadherin (*p < 0.05 vs control group). (**C)** The percentages of the positive expression of PCNA (*p < 0.05 vs control group). H&E and immunohistochemistry staining with magnification of 200 × .

To further explore the potential mechanism of X-PDT treatment, we performed H & E and immunohistochemical staining on tumor tissue in each group. The images (Fig. 9A) showed marked inflammatory infiltration with necrosis in tumor tissue from Cu-Cy and X-PDT groups. In addition, E-cadherin and PCNA, essential biomarkers of tumor cell proliferation and migration, expression from the Cu-Cy group and X-PDT group (Fig. 9B and C) showed significant differences in comparison to the control group. The positive rate of the E-cadherin and PCNA expression in the X-PDT group was 44.3% and 24.6%. On the other hand, the positive rate in the control group was 14.0% and 44.0%. These results further confirmed the conclusion from Fig. 3, Fig. 4 that Cu-Cy NPs based X-PDT inhibited the cell proliferation and cell migration.

## 4 Discussion

For clinical applications, it is important to demonstrate that NPs can be activated to exert antitumor efficacy using clinically relevant tumor models [20]. Until now, X-PDT has been shown primarily *in vitro* or with subcutaneous tumor models *in vivo* under laboratory environments. The present study is different from previous studies in that we mimicked deeply located tumor models by placing a layer of pork (around 2 cm thickness) between tumor cells or tumor tissues and an X-ray generator and built a deeply-located model of VX2 liver tumor in rabbits. Furthermore, we chose a clinical linear accelerator as an X-ray generator instead of the experimental X-ray machine to mimic the traditional RT therapy. The above changes were performed in this study in order to further explore the effectiveness of X-PDT based Cu-Cy NPs in deeply located tumor models under the clinical environment. According to the previous studies, *in vitro* studies on different cancer cells revealed noticeable cell apoptosis using Cu–Cy when excited by X-rays [[12], [13], [14]] and Chen's group also demonstrated their X-PDT performance for *in vivo* cancers [[11], [12], [13], [14]]. Most interestingly, the present findings are in accordance with previous studies, indicating that X-PDT based on Cu-Cy NPs not only could improve the efficiency of RT for the treatment of deeply seated tumors but also has a promising clinical application prospect.

Metastasis, which is considered as a leading cause of cancer-related death, is a complex phenomenon involving various steps, such as cancer cell dissemination, invasion, migration, intravasation, extravasation, and colonization [26]. Tumor migration is a key process and contains several steps, including the epithelial-to-mesenchymal transition process, by which epithelial cells can lose their adhesive capability to adhere to adjacent cells and acquire mesenchymal phenotype [[27], [28], [29], [30], [31]]. The transmembrane protein E-cadherin is one of the main proteins that form intercellular connections between epithelial cells, so it is considered as a biomarker of epithelial phenotype [[31], [32], [33]]. Thus, there is a negative relationship between E-cadherin expression and tumor migration [33]. Cell proliferation is considered to play a key role in the development of tumor growth. PCNA [34], a highly conserved nuclear protein of DNA polymerase-delta, has been widely used as a specific marker to evaluate tumor cell proliferation [35]. Decreased expression of PCNA in the tumor tissue suggests the low proliferative activity of tumor cells. For X-PDT, it is very important to study its effects on tumor cell migration in addition to its anticancer effect owing to the induction of oxidative stress and ROS-facilitated cell killing in cancer as the X-PDT mediated reactive oxygen species (ROS) are closely related to tumor cell migration and invasion [36]. In addition, the generation of ROS during X-PDT may increase hypoxia [37] that may stimulate cancer cell migration and invasion [[37], [38], [39]].

Several studies show that ROS adds to tumor malignancy by partaking in molecular and cellular processes in signaling pathways and in the transcriptional activities that induce cell migration, invasion, and Epithelial-mesenchymal transition (EMT). Furthermore, ROS play a vital role in the migration and invasion of cancer cells [36,40,41]. Higher ROS levels frequently overload antioxidant systems, resulting in oxidative stress. Various oxidative stress levels are found to induce distinct results in cancer cells. Mild oxidative stress initiates cell signaling mechanisms, such as proliferation, invasion and migration, while high oxidative stress can cause cell death. It has been found that ROS are important for various integrin-mediated cellular responses, such as adhesion, cytoskeleton organization, migration, proliferation, and differentiation. EMT is vital for the migration and invasion of several cancerous cell. Some findings have indicated the part ROS plays in the creation of EMT in cancer, while at the same time ROS can initiate Snail, leading to EMT for cancer migration [36,41].

Hypoxia is a key factor in the tumor microenvironment (TME) that regulates various characteristics of cancer, including angiogenesis, invasion, EMT, stemness, and immune evasion [39,42,43]. HIF-1, being an oxygen-dependent transcriptional activator, performs a vital role in tumor development as well as in metastasis. Also, the increased expression level of HIF-1a is found under hypoxic conditions [44]. EMT has been found as an important event in the metastatic cascade, allowing cells to proliferate [39]. Furthermore, HIF-1 supports EMT via direct regulation of EMT-related proteins, including TWIST during the EMT process [39,41]. Also, E-cadherin can play a vital role in tumor invasion and metastasis [44]. In healthy tissue, E-cadherin and b-catenin develop an E-cadherin/b-catenin complex, thereby maintaining cell polarity and organizing structural stability. On the other hand, E-cadherin expression is reduced inside tumors, which reduces the bonding properties of cells and boosts proliferation ability, which in turn leads to cell migration. Furthermore, E-cadherin is correlated with hypoxia-induced tumor cell migration and invasion. Several studies show that ROS induces HIF-1a expression [41,44]. As a consequence, ROS promotes EMT and tumor migration [41].

To summarize, the X-PDT based on Cu-Cy NPs that produce ROS may cause oxidative stress and cell injury or cell killing directly. Furthermore, ROS can affect cell migration or invasion. As discussed above, the production of ROS may further promote the formation of hypoxia that may affect the tumor environment and cell migration. Notably, our results demonstrate that under X-ray irradiation, Cu-Cy NPs generate a significant ROS level, thereby resulting in the direct destruction of cancer cells. Apart from the ROS production, Cu-Cy NPs upon X-ray can effectively induce an anti-tumor immune response. Taken together, Cu-Cy NPs can simultaneously enable oxidative therapy, radiotherapy, and immunotherapy on cancer treatment [14].

**Fig. 10:**
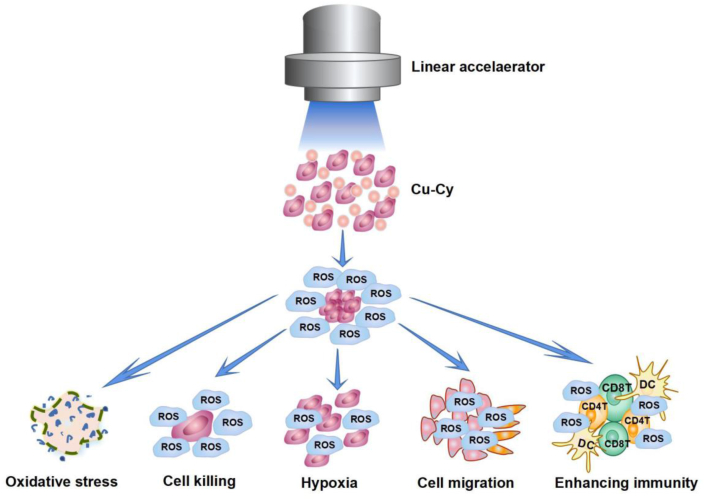
The possible influences of X-PDT on cells: the main effects from ROS including oxidative stress, cell killing by direct injury, hypoxia formation due to the consumption of oxygen, possible induction of cell migration, and invasion as well as enhancing immunity.

The possible effects of Cu-Cy based X-PDT are summarized in Fig. 10. The possible induction of cell migration is considered a ‘negative effect’ from the treatment. In this contribution, we particularly paid attention to the influence of the X-PDT on tumor migration and the results showed that X-PDT based on Cu-Cy NPs could inhibit tumor cell proliferation and migration via modulating PCNA and E-cadherin. That is the ‘negative effect’ is not observed from the X-PDT, which indicates that the ROS from the interaction of X-ray with Cu-Cy NPs is mainly used for cancer cell oxidative stress, killing, and immunity enhancement that are beneficial to cancer treatment.

The toxicity of NPs from their size and unique physiochemical properties has been considered a limitation for their wide-spread uses [45]. In this work, we have performed several assays to validate the cytotoxicity of Cu-Cy NPs. The cytotoxicity assays came from three levels of biological evaluation, including cell (CCK8), tissue (pathological examination), and animal (ethological changes). The results have demonstrated that Cu-Cy NPs at the concentration of 100 mg/L did not show significant cytotoxicity *in vitro*. Furthermore, no damages to major organs were observed, and no acute toxicity reaction occurred *in vivo*.

Another interesting topic is the change of hypoxia-inducible factors in the treated tumors. HIF-1 is a heterodimer composed of an O_2_-regulated HIF-1α subunit and a constitutively expressed HIF-1β subunit. Under normoxic conditions, HIF-1α is continuously synthesized and degraded. However, under conditions of reduced O_2_ availability (hypoxia), HIF-1α degradation is inhibited, then the protein accumulates, dimerizes with HIF-1β, binds to *cis*-acting hypoxia response elements in target genes, and recruits coactivator proteins, which leads to increased transcription. O_2_-dependent degradation of HIF-1α is triggered by binding of the von Hippel-Lindau tumor-suppressor protein (VHL), which interacts with the protein Elongin C, thereby recruiting an E3 ubiquitin-protein ligase complex that ubiquitinates HIF-1α and targets it for degradation by the 26S proteasome [46].

Hypoxia is the best-characterized mechanism of HIF activation in tumors. Consistent with tumor hypoxia as a mechanism of HIF activation, HIF protein is commonly detected in perinecrotic regions of sporadic tumors and overlaps with staining for known hypoxic markers. Jiang et al. [47] have quantitated HIF-1 DNA-binding activity and protein levels of the HIF-lα and HIF-lβ subunits in human HeLa cells exposed to O_2_ concentrations ranging from 0 to 20% in the absence or presence of 1 mM of KCN to inhibit oxidative phosphorylation and cellular O_2_ consumption. HIF-1 DNA-binding activity, HIF-lα protein, and HIF-lβ protein each increased exponentially as cells were subjected to decreasing O_2_ concentrations, with a half-maximal response between 1.5 and 2% O_2_ and a maximal response at 0.5% 0_2_, both in the presence and absence of KCN. The HIF-1 response was greatest over O_2_ concentrations associated with ischemic/hypoxic events *in vivo*. These results provide evidence for the involvement of HIF-1 in O_2_ homeostasis and represent a functional characterization of the putative O_2_ sensor that initiates hypoxia signal transduction leading to HIF-1 expression.

During the treatment of X-ray induced oxidative therapy, O_2_ is consumed for the formation of ROS. In this case, it is expected that the HIF-lα protein, and HIF-lβ protein would increase in the treated tumors. However, recent studies indicate that mitochondrial ROS also plays an important role in regulating HIF protein levels under hypoxia [48,49]. Multiple groups have observed that genetic and chemical inhibition of the mitochondrial electron transport chain and ROS production results in decreased HIF stability under hypoxic conditions. This means the interplay of oxygen concentration or hypoxia and ROS on HIF-1 is a complicated manner [48]. More research is needed to figure out the effects of X-ray induced oxidative therapy on HIF-1 and its outcomes on the therapeutic efficacy.

One of the advantages of using copper-cysteamine is that it is nanoparticles. So, it accumulates more in cancer cells than in normal cells by EPR effect. More importantly, Cu-Cy nanoparticles do not have sunlight toxicity, which will be described in our forthcoming papers. Traditional sensitizers like porphyrins have sunlight toxicity, which means patients treated with porphyrins are needed to stay in dark for about 1 week. However, patients treated with Cu-Cy can walk outside freely as Cu-Cy does not produce ROS under sunlight. Synergistic therapy is a feasible route for improving the therapeutic effect and survival rate of patients with cancer. Notably, therapeutic effects are significantly enhanced when various cancer treatments are combined through intelligent models. Cu-Cy can be excited by different sources, including microwave. If Cu-Cy is activated by X-ray and microwave, the issue of hypoxia can also be solved as heat can solve the issue of hypoxia. So, there are many advantages of Cu-Cy over traditional photosensitizers.

In addition, Copper, just like Fe, can induce Fenton Reaction to produce ROS, would this be considered as an advantage of this material. In tumor acidic environment, copper ions are released from Cu-Cy to induce Fenton reaction to form ROS for tumor cell destruction, while in the neutral conditions for normal cells, copper ions cannot be released from Cu-Cy and no Fenton reactions occur. So, Cu-Cy nanoparticles can selectively kill cancer cells but not healthy cells. This has been discussed in our recent publication [50]. All these indicate the potential of Cu-Cy NPs for practical applications.

The safety and pharmaceutic dynamics of these nanoparticles have been studied and reported in our previous paper [6]. In our pilot studies, we did not see any serious toxicities [6]. Similarly, we focused on animal welfare in this study, such as a comfortable environment, the observation of abnormal behavior, and the changes in animal body weight. Yes, more physiological and biochemical tests should be conducted in the aspect of reflecting the whole-body health conditions. In addition, more work is be ing done to the extention of these materials for more applications [51].

## 5 Conclusions

In conclusion, we have mimicked the clinical conditions for Cu-Cy mediated PDT and our observations show that Cu-Cy NPs have a promising clinical application prospect in X-PDT to improve the efficiency of RT for the treatment of deep-seated tumors. More interestingly, our studies found that Cu-Cy mediated PDT can effectively inhibit tumor cell proliferation and migration. These new observations further facilitate that X-ray induced PDT is a promising modality to fight against cancers.

## CRediT author statement

**Nil Kanatha Pandey, Jiayi Liu, Ya Li, and Eric Amador**: Methodology, Synthesis, Cell and Animal Studies, and Characterization; **Lingyun Wang, Xiangyu Chen, Taili Chen, Feiyue Liu and Wei Chen**: Conceptualization, Instruction, Funding and Writing; **Enhua Xiao**: Funding and instruction, **Xiangyu Chen**: Data analysis and editing.

## Declaration of competing interest

The authors declare that they have no known competing financial interests or personal relationships that could have appeared to influence the work reported in this article.
